# Impact of Pulsed Electric Fields on the Volatile Compounds Produced in Whole Onions (*Allium cepa* and *Allium fistulosum*)

**DOI:** 10.3390/foods7110183

**Published:** 2018-11-07

**Authors:** Rajkumar Nandakumar, Graham T. Eyres, David J. Burritt, Biniam Kebede, Michelle Leus, Indrawati Oey

**Affiliations:** 1 Department of Food Science, University of Otago, P.O. Box 56, Dunedin 9054, New Zealand; nraj.sathya@hotmail.com (R.N.); graham.eyres@otago.ac.nz (G.T.E.); biniam.kebede@otago.ac.nz (B.K.); michelle.leus@otago.ac.nz (M.L.); 2 Department of Botany, University of Otago, P.O. Box 56, Dunedin 9054, New Zealand; david.burritt@otago.ac.nz; 3 Riddet Institute, Private Bag 11 222, Palmerston North 4442, New Zealand

**Keywords:** pulsed electric field, onions, HS-SPME-GCMS, volatile sulfur compounds

## Abstract

The objective of this research was to investigate the effect of pulsed electric field (PEF) processing on the volatile compounds produced in onion cultivars. The changes in the volatile compounds of onions were assessed comparing results observed while measured immediately and 24 h after PEF treatment using headspace solid-phase microextraction gas chromatography-mass spectrometry (HS-SPME-GC-MS). Significant increases in the concentrations of propanethial S-oxide, propenyl propyl thiosulfinate, 2-methyl-2-pentenal, dipropyl disulfide, propenyl propyl disulfide, methyl propyl disulfide, and methyl propenyl disulfide were observed immediately after PEF treatment. The concentrations of propenyl propyl thiosulfinate, dipropyl disulfide, methyl propyl disulfide, dipropyl trisulfide, methyl propyl trisulfide, and propenyl propyl trisulfide increased after 24 h compared to initial concentrations. It is postulated that these changes are due to PEF-induced cell permeabilisation that facilitates enzyme-substrate reactions after the PEF treatment.

## 1. Introduction

Pulsed electric field (PEF) technology has stimulated intense research as an alternative processing technique to achieve cellular disruption since the 1960s [1,2]. This processing applies short (micro- to milli-seconds) pulses of high voltage across food materials placed between electrodes [3,4]. PEF processing at electric field strengths lower than 2 kV/cm can modify the structure and texture of plant tissue, which can enhance mass transfer, thereby facilitating extraction of phytochemicals and improving the accuracy and energy efficiency of cutting and slicing [5,6]. However, most of these phenomena have been studied on mechanically fragmented tissue sections such as tissue disks [7,8,9], tissue slices [8,10], tissue cubes [11], and tissue cylinders [12,13]. PEF treatment has been shown to produce non-uniform effects on the microstructure of intact tubers (e.g., potato and kumara) [14,15]. Thus far, limited information is available on how PEF treatment influences the volatile profile of intact plant materials.

Onions (*Allium cepa* and *Allium fistulosum*) are commonly used for culinary purposes [16] and are rich in *trans*-S-propenyl cysteine sulfoxide (PECSO), S-propyl cysteine sulfoxide (PCSO), and S-methyl cysteine sulfoxide (MCSO). These compounds act as volatile flavor compound precursors [17,18]. The volatile compounds in onions are produced when cell damage occurs facilitating the mixing of the enzyme alliinase and the flavor precursors, which are present in different compartments of the cell, namely in vacuole and cytoplasm, respectively [19].

In the literature, the volatile compounds in onions are typically characterized on completely disrupted tissue, where the enzymatic reactions have been allowed to occur completely over a defined time period. This study aimed to assess the changes in the volatile profile in intact onions immediately after PEF treatments and 24 h after PEF treatment in order to understand the impact of PEF processing and storage on an onion’s physiochemical properties.

## 2. Materials and Methods

### 2.1. Chemicals and Reagents

Potassium dihydrogen phosphate (KH_2_PO_4_), dipotassium hydrogen phosphate (K_2_HPO_4_), mannitol, sodium chloride (NaCl), and fenchyl alcohol were purchased from Sigma Aldrich (St. Louis, MO, USA). Methanol (LiChrosolv liquid chromatography grade) was purchased from Merck (Darmstadt, Germany).

### 2.2. Onion Sample Preparation

Three onion varieties, namely Yellow Sweet Spanish bulbs (*Allium cepa*), Red Bunching, and Ishikura White Bunching (*Allium fistulosum*), were cultivated under controlled conditions in the field of Department of Botany (University of Otago, Dunedin, New Zealand) and hand-harvested at the age of 20 weeks. Immediately after harvest, the onions were visually screened for any damage or bruises and sorted based on their diameter (13–20 mm) as a measure of maturity. All onion samples were gently cleaned of any dirt and any papery outer scale. The samples were stored at 4 °C and 90–100% relative humidity under subdued light and used within 24 h.

Before PEF treatment, onion samples were cut to achieve a total length of 7 cm (0.5 cm of root and 6.5 cm of shoot regions) to fit inside the PEF treatment chamber. A randomized paired experimental design was used for each cultivar and PEF treatment in order to minimize the variability between the two-time sampling points, i.e., immediately (T0) and 24 h (T24) after PEF treatment. Two matching onions were paired (one for T0 and the other for T24), weighed and positioned side by side inside the chamber using a non-conductive sample holder. The roots were facing the high voltage electrode of the chamber. The onion samples were fully immersed in potassium phosphate buffer (10 mM, pH 7.0, conductivity 1.68 ± 0.73 mS), avoiding air bubbles, and made up to a constant total weight of sample and buffer of 350 g. The same sample preparation procedure was mimicked for the untreated (control) onion samples, at T0 and T24.

### 2.3. PEF Treatment

PEF equipment used in this investigation was an ELCRACK^®^ HVP-5 (German Institute of Food Technologies, Quakenbrück, Germany) in batch configuration. The treatment chamber (400 mL capacity, 100 mm length × 80 mm width × 50 mm height) consisted of two stainless steel electrodes of 5 mm thickness separated by a distance of 80 mm.

In this study, electric field strengths of 0.3 (low PEF), 0.7 (medium PEF), and 1.2 kV/cm (high PEF) using a pulse width of 20 µs, a pulse frequency of 50 Hz, and a specific energy of 5 kJ/kg were applied for comparison to untreated (control) samples. All PEF treatments were carried out at room temperature (20 ± 2 °C) and the temperature changes due to PEF treatments were found to be negligible. All PEF treatments were conducted in a randomized order and repeated using eight independent samples as replicates.

Immediately after PEF treatment, both samples were removed from the buffer. The T0 sample was packed in aluminum foil, snap-frozen in liquid nitrogen, and stored at −80 °C. The T24 sample was placed on wet paper towels and kept at 4 °C for 24 h, snap-frozen, and stored at −80 °C as per the T0 samples.

The frozen onion samples were subsequently ground under liquid nitrogen into a fine powder using a mortar and pestle. The frozen onion powder was stored at −80 °C in sealed Eppendorf tubes until analysis.

### 2.4. Headspace SPME-GC-MS

Frozen onion powder (0.5 g) was mixed with 2 mL of 90% methanol in a 20 mL headspace vial. The sample was swirled and left to stand in an ice slurry for 10 min to inactivate the enzyme activity. Saturated NaCl solution (8 mL) and fenchyl alcohol (internal standard (IS), 100 µL of 10 ppm) were then added to the headspace vial and sealed with a PTFE-coated silicon septum screw cap. Samples were then loaded on to the auto sampler (PAL RSI 85, CTC Analytics, Zwingen, Switzerland) for analysis.

Volatile compounds were extracted using a divinylbenzene/carboxen/polydimethylsiloxane (DVB/CAR/PDMS; 50/30 µm) SPME fiber (Supelco, Sigma-Aldrich) for 30 min at 40 °C. Volatile compounds were analyzed using an Agilent 6890N gas chromatograph equipped with a VL MSD 5975B mass spectrometer (Agilent Technologies, Santa Clara, CA, USA). Separations were achieved on a Zebron ZB-WAX capillary column (60 m × 0.32 mm ID, 0.50 μm d.f., Phenomenex, Torrance, CA, USA) using helium as the carrier gas at 1.0 mL/min constant flow. The sample desorption was performed for 5 min at 240 °C in the GC injection port (split/splitless inlet) operated in splitless mode for 2 min. The oven temperature was held at 40 °C for 3 min, increased at 5 °C/min to 120 °C, and then increased at 10 °C/min to 240 °C with a hold time of 5 min (total run time 38 min).

The MS was operated at a source temperature of 230 °C, interface temperature of 150 °C, using electron impact (EI) mode with electron energy at 70 eV. All the analyses were carried out in a full scan mode, scanning in the range of 30–250 amu at a scan rate 1.56 u/s (6.1 scans/s). MSD ChemStation software (version D.03.00.611, Agilent Technologies, Santa Clara, CA, USA) was used for peak analysis of the volatile compounds. Compounds were tentatively identified by comparing the mass spectra to the National Institute of Standards and Technology (NIST 2014) database. The extracted ion count of the detected compounds was manually integrated, using specific mass ions. Linear retention indices were calculated to support the identification of the compounds. Samples were analyzed in a randomized order, blocked by biological replicates.

### 2.5. Statistical Analysis

The experimental data was collected from three onion cultivars, each inclusive of control and three PEF treatments in a randomized order and replicated using eight independent samples.

Principal component analysis (PCA) was carried out to investigate the relationship between the volatile compound concentration and the time period of analysis for each control and PEF treatment intensity, using Solo (version 8.2, 2017, Eigenvector Research, Wenatchee, WA, USA).

The data was checked for outliers using a two tailed (Kolmogorov–Smirnov) normality test, with a significance level of *p* ≤ 0.05, using The Unscrambler X (CAMO Analytics, Manolia, TX, USA). The data was then analyzed using two-factor analysis of variance (ANOVA) (treatment and time), with a significance level of *p* ≤ 0.05, using XL STAT (Addinsoft, New York, NY, USA). Significantly different means were determined using Fisher LSD’s post hoc comparison testing. Mean peak areas (extracted ion) and standard errors of the mean were calculated using eight independent replicates.

## 3. Results and Discussion

### 3.1. Identification and Characterisation of Volatile Compounds

A total of 14 volatile compounds were detected in both control (untreated) and PEF-treated samples, in addition to the internal standard and methanol (Table 1). The volatile compounds detected in the onion cultivars can be categorised into three chemical classes: alkanes (nonane, decane, and undecane), aldehyde (2-methyl-2-pentenal), and the sulfur-containing compounds (propanethial s-oxide, methyl propyl disulfide, dipropyl disulfide, propyl propenyl disulfide, methyl propyl trisulfide, dipropyl trisulfide, propenyl propyl thiosulfinate, and propenyl propyl trisulfide). These dominant sulfur-containing compounds are responsible for the characteristic onion flavour [18,20].

In the control samples, low concentrations of volatile compounds were detected. This could be due to compounds generated as a result of cutting during sample preparation. Upon PEF treatment, the concentrations of these compounds increased significantly (*p* < 0.05) immediately after PEF treatment and after 24 h incubation (Tables S1–S4). These results demonstrate that the volatile compounds are generated upon the application of PEF treatment.

### 3.2. Evaluation of PEF Treatment Intensities and Incubation Time on the Volatile Compounds

**Figure 1:**
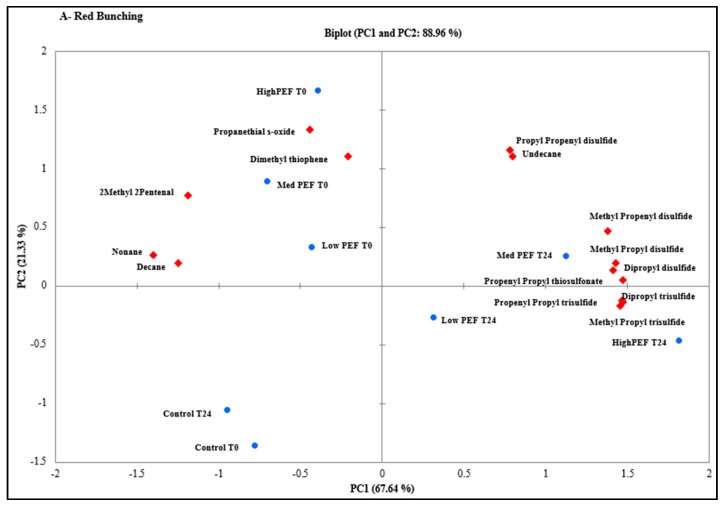

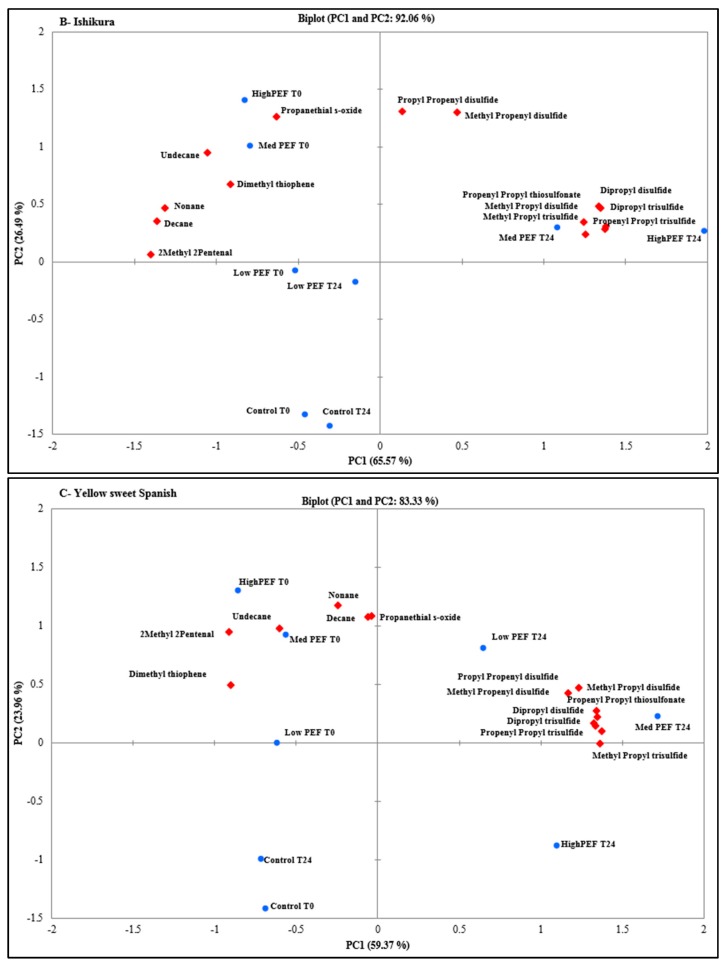
PCA biplot of first and second principal components of PEF treatment intensities and volatile compounds produced by (**A**) Red Bunching, (**B**) Ishikura, and (**C**) Yellow Sweet Spanish bulb onions, analyzed at 0 h and after 24 h. Each data point represents independent onion sample (*n* = 8).

Principal components analysis (PCA) was utilised to visualise the differences in the headspace concentrations of the detected volatile compounds due to different PEF treatment intensities and incubation time. The PCA biplots for each of the three onion cultivars explained between 83% and 92% of the variance on the first two principal components (PCs) (Figure 1). The trends explained by the PCs for the three cultivars studied were found to be similar. The first trend explained by PC1 is the effect of incubation time on the PEF-treated samples. The T0 samples are negatively correlated (left) on the PC1 axis, whereas the T24 samples are positively correlated (right). The magnitude of this effect was found to be greater at medium- and high-PEF intensities. The second trend explained by PC2 is the effect of PEF treatment intensities at T0. The two control samples (T0 and T24) are both negatively loaded on PC2, and an increase in electric field strength moved the scores of PEF treatment intensities towards the positive loading of PC2 for T0 samples.

The impact of PEF treatment on volatile compounds was found to be different between the onion cultivars studied. The differences could have been caused due to their difference in the relative concentration of volatile precursors, which is based on the genotype and the growth environment (e.g., sulfur supply) [23]. In the Red Bunching onions, the variation along PC1 (67.6%) was found to be correlated with the incubation time upon PEF treatment (Figure 1A), whereas the PEF treatment intensities were discriminated from each other along PC2 (21.3%).

The concentration of PSO, nonane, decane, 2M2P, and DMT were associated with the medium- and high-PEF treatment intensities for the T0 samples. In contrast, the higher concentrations of sulfur-containing compounds (thiosulfinates, disulfides, and trisulfides) were found to be associated with the medium- and high-PEF treatment intensities at T24. The variations observed in both PCs of the Ishikura onions were found to follow a similar trend for the Red Bunching onions. The variation along PC1 (65.6%) was correlated with incubation time upon PEF treatment (Figure 1B) and the PEF treatment intensities were discriminated from each other along PC2 (26.5%). The concentration of PSO, alkanes (nonane, decane, and undecane), 2M2P, and DMT were highly correlated to the medium- and high-PEF-treated samples at T0; in the top left quadrant (negative PC1 and positive PC2). In contrast, the concentration of the volatile sulfur compounds (disulfides and trisulfides) were found to be proportionally higher in the medium- and high-PEF-treated samples at T24; in the top right quadrant (positive on both PCs).

A different trend was observed for Yellow Sweet Spanish bulbs (Figure 1C). The variations between the PEF treatment intensities were found to be highest between the control and medium-PEF-treated samples, with PC2 explaining 24% of the variance. The trend between PEF-treated samples analysed at T0 and T24 remained the same, being principally explained on PC1 (59.4% explained variance). The concentration of PSO, alkanes (nonane, decane, and undecane), 2M2P, and DMT were found to be highly associated with the medium-PEF treatment intensity at T0, whereas the concentration of disulfides and trisulfides were found proportionally higher in concentration upon medium-PEF-treated samples at T24.

These PCA results have demonstrated a direct correlation between the PEF treatment intensities and the overall concentration of volatile compounds in each onion cultivar. Moreover, the impact of incubation time upon PEF treatment was found to play an important role in the final concentration of volatile compounds, across all onion cultivars.

### 3.3. Effect of PEF Processing Intensities on the Volatile Concentrations

**Figure 2:**
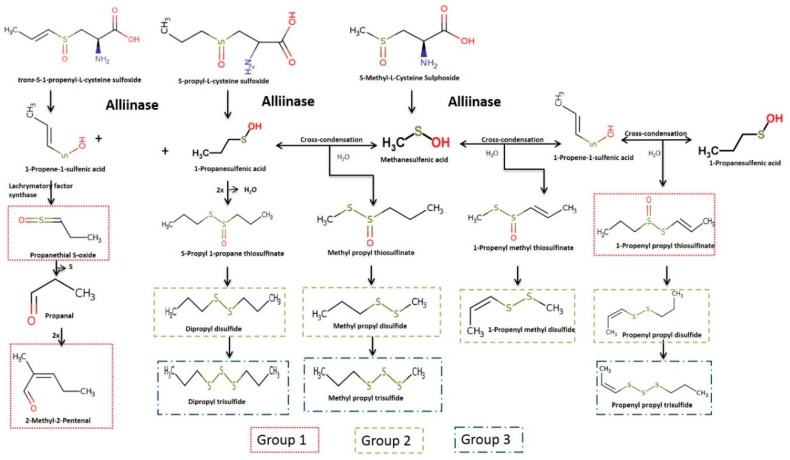
Enzymatic production cascade of onion flavour compounds. Self and cross condensation products of propenyl, propyl, and methyl cysteine sulfoxides. Some reaction products are not shown for clarity. Adapted from [24,25].

The effect of PEF treatment had a significant (*p* ≤ 0.05) effect on the concentration of volatile compounds (Table S1). The concentration of volatile compounds detected in the control samples was found to be similar across the two-time points analysed. However, the concentrations of volatile compounds detected in the PEF-treated samples were found to be dependent on the applied electric field strength at each time point (treatment effect, Table S1). The detected volatile compounds have been grouped into three groups, based on their position in the enzymatic reaction cascade (Figure 2).

**Figure 3:**
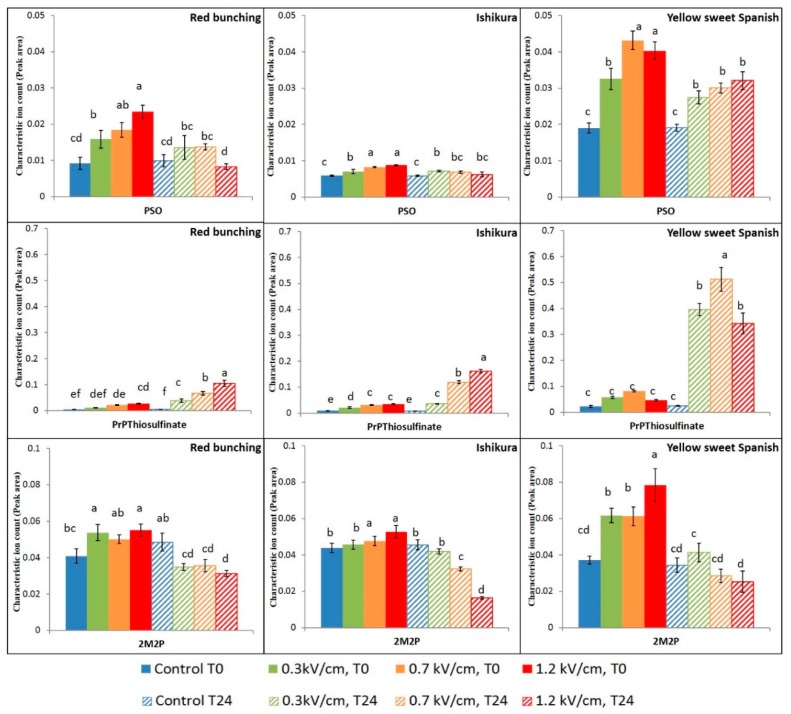
Mean (*n* = 8) ion counts of propanethial S-oxide (PSO), propenyl propyl thiosulfinate (PrPthiosulfinate), and 2-methyl-2-pentenal (2M2P) in control and PEF-treated onion samples. Peak areas have been standardised to the internal standard. Bars with different letters indicate statistically significant difference (Fisher’s LSD, *p* < 0.05). Error bars represent standard error of the mean.

**Figure 4:**
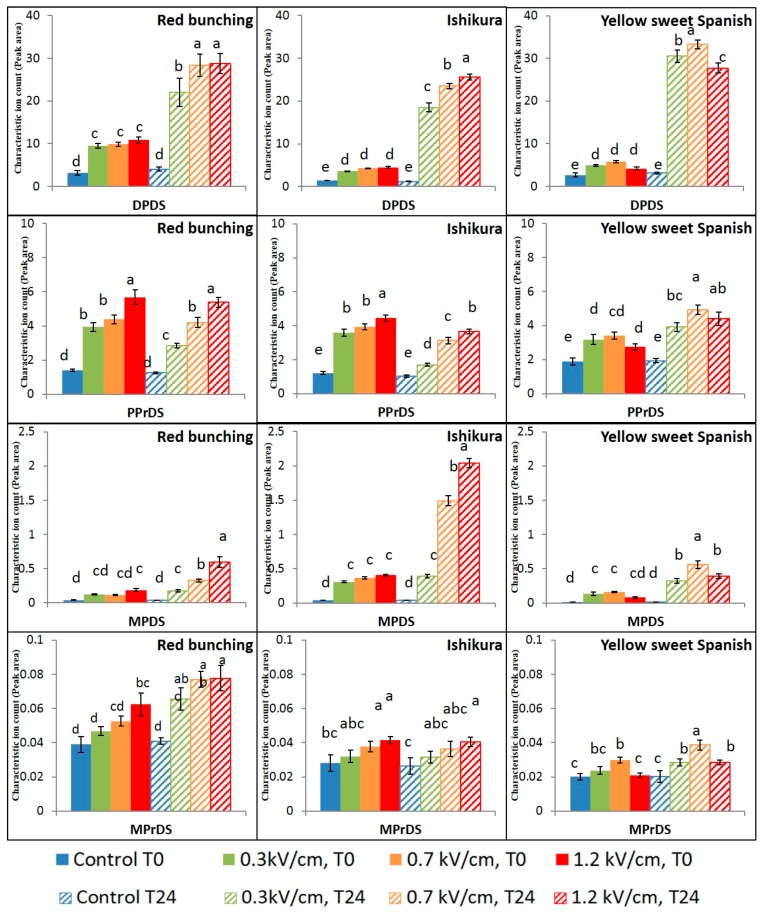
Mean (*n* = 8) ion counts of methyl propyl disulfide (MPDS), dipropyl disulfide (DPDS), Propyl propenyl disulfide (PPrDS), and methyl propenyl disulfide (MPrDS) in control and PEF-treated onion samples. Peak areas have been standardised to the internal standard. Bars with different letters indicate statistically significant difference (Fisher’s LSD, *p* < 0.05). Error bars represent standard error of the mean.

**Figure 5:**
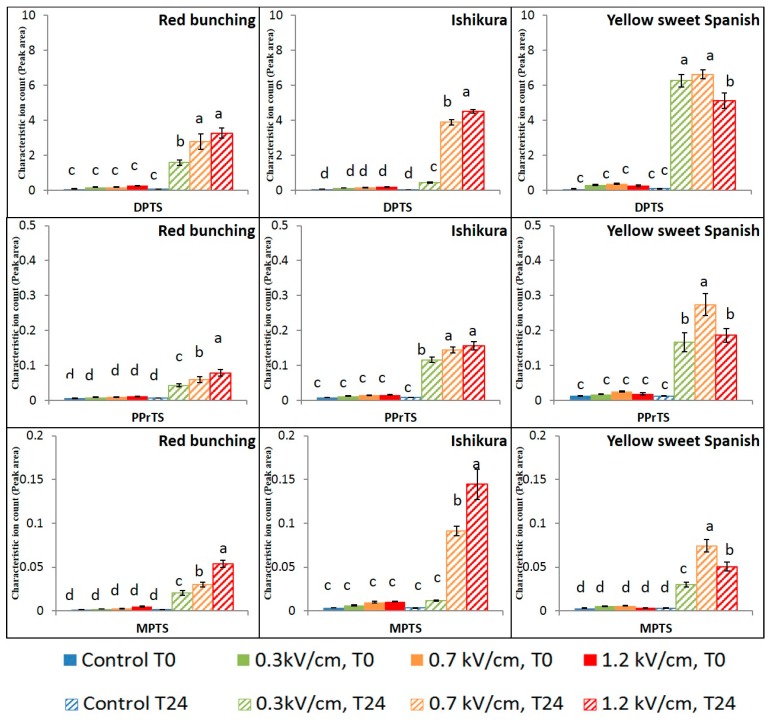
Mean (*n* = 8) ion counts of dipropyl trisulfide (DPTS), propyl propenyl trisulfide (PPrTS), and methyl propyl trisulfide (MPTS) in control and PEF-treated onion samples. Peak areas have been standardised to the internal standard. Bars with different letters indicate statistically significant difference (Fisher’s LSD, *p* < 0.05). Error bars represent standard error of the mean.

Group 1 contains the highly reactive propanethial S-oxide, 2-methyl-2-pentenal, and an intermediate compound, propenyl propyl thiosulfinate (Figure 3). Group 2 contains the disulfide compounds, namely dipropyl disulfide, propenyl propyl disulfide, methyl propyl disulfide, and methyl propenyl disulfide (Figure 4). Group 3 contains the trisulfide compounds, namely dipropyl trisulfide, propenyl propyl trisulfide, and methyl propyl trisulfide (Figure 5).

The propanethial s-oxide (PSO) in onions is interesting due to its tear-inducing nature (lachrymatory factor). In the onion cultivars tested, PEF treatment intensities had a considerable effect on the concentrations of PSO measured at T0. The concentration of PSO increased with greater applied electric field strengths (>0.3 kV/cm) for all three cultivars (Figure 3). Asavasanti et al. [7] reported that the critical electric field strength (*Ec*) required to rupture the onion cell and tonoplast membrane was between 0.2 and 0.33 kV/cm, supporting the current result. Increasing electric field strength resulted in enhanced cell membrane permeabilisation facilitating the occurrence of enzymatic reactions, such as the reactions between alliinase and alkenyl cysteine sulfoxides (precursor), resulting in high concentrations of PSO being observed.

After 24 h following PEF treatment, the impact of PEF treatment on PSO concentrations was found to be similar or lower than the impact at T0. The application of PEF treatment intensities has resulted in the enzymatic reaction, generating 1-propenesulfenic acid as the first intermediate compound, which is further catalysed by lachrymatory factor synthase (LFS) to form propanethial s-oxide (Figure 4) [26,27]. The reduction in the concentration of PSO during 24 h incubation after PEF treatment, could be due to hydrolysis or loss due to its high vapour pressure (41.2 ± 0.2 mmHg at 25 °C) [28].

The alkyl thiosulfinates are rapidly formed via self- or cross-condensation of sulfenic acids upon enzymatic initiation of the reaction cascade [25]. The concentration of propenyl propyl thiosulfinate, detected at T0, was found to increase with an increase in electric field strength in the spring onions (Red Bunching and Ishikura) (Figure 5). However, no effect of PEF treatment was observed in the Yellow Sweet Spanish bulbs at T0. The differences observed could be due to the physiological properties of the cultivar and the maturity of the bulb tissue.

After 24 h incubation following PEF treatment, the concentration of thiosulfinates significantly increased in all the onion varieties. The highest relative concentration of thiosulfinates upon PEF treatment was obtained at different treatment intensities in the onion varieties. The highest concentration of thiosulfinate in the spring onions was obtained after 24 h incubation with a high-PEF treatment. However, the highest concentration of thiosulfinate in the Yellow Sweet Spanish bulbs was obtained upon 24 h incubation with a medium-PEF treatment. Application of high-PEF treatment intensity resulted in a relatively low concentration of thiosulfinate after 24 h of the PEF treatment in the bulbs. The relative drop in the concentration of thiosulfinate in the Yellow Sweet Spanish bulbs could be due to the extensive disintegration of the plasma and vacuole membrane resulting in the leaching of the alkenyl cysteine sulfoxides from the cell cytoplasm, thus reducing the available precursor substrate for the reaction [29,30].

2-Methyl-2-pentenal (2M2P) was the only aldehyde detected in the onion samples in this study. 2M2P is a self-condensation product formed from propanal (Figure 2) and is reported to contribute a fruity flavour to onion samples [31]. PEF treatment increased the concentration of 2M2P immediately after the treatment, and the concentration of 2M2P was afterwards found to decrease across all onion cultivars after 24 h incubation. The reduction in the concentration of 2M2P was found to be significantly higher upon the application of medium- and high-PEF treatment intensities. This reduction in the concentration of 2M2P after 24 h incubation after PEF treatment could be due to evaporation occurring during incubation.

The application of PEF treatment significantly (*p* ≤ 0.05) increased the concentration of disulfide compounds, categorised in Group 2 (Figure 4). Block [19] reported that the disulfides are formed upon the rearrangement of the alkyl thiosulfinates, which further depend on the degree of cell damage and time duration taken for the enzymatic reaction to occur. The impact of PEF treatment intensities on the concentration of disulfide compounds was found to be in the order of dipropyl disulfide (DPDS) followed by propenyl propyl disulfide (PPrDS), methyl propyl disulfide (MPDS) and finally methyl propenyl disulfide (MPrDS). The impact of PEF treatment intensities was found to be similar for the concentration of DPDS and MPDS, at both time points measured (Figure 4). The concentration of DPDS and MPDS increased (2–3 fold) immediately after PEF treatment, irrespective of the electric field strength. This implies that the reaction cascade initiated upon PEF treatment has not reached completion at T0. However, the concentration of DPDS and MPDS increased enormously (7–30 fold) after 24 h of incubation and was dependent on the applied electric field strength.

The application of PEF treatment was found to have a different impact on the concentration of the PPrDS and MPrDS (Figure 4). The concentration of PPrDS and MPrDS increased with elevating electric field strength, measured at both time points across all onion varieties. There was no significant difference in the concentration of PPrDS and MPrDS measured between the two time points. This implies that the enzymatic reaction had almost reached completion at T0 and no further increase in the concentration of PPrDS and MPrDS occurred during the incubation period. The application of high electric field strength resulted in the highest concentration of DPDS and MPDS in the spring onions, whereas the highest concentration of DPDS and MPDS in the Yellow Sweet Spanish bulbs was achieved after 24 h with a medium-PEF intensity treatment. The maximum concentrations of disulfide compounds could also be attributed to the relative concentration of their substrates, which differs between onion cultivars [32,33]. Overall, it could be inferred that the impact of PEF treatment had a significant effect on the concentrations of disulfides and was based on the applied electric field strength dependent on the onion varieties tested.

The application of PEF treatment was found to be insignificant on the three trisulfide compounds immediately after PEF treatment (T0, Figure 5). However, after 24 h incubation following PEF treatment, the concentrations of trisulfides were dependent on the electric field strength applied. The trisulfide compounds are formed upon the rearrangement of the respective alkyl disulfide compounds, along with a monosulfide substituent. Since the trisulfides are formed later in the reaction cascade (Figure 2), more time was required for the enzyme initiated reaction cascade to reach completion [34] and thus a difference to be measured. It was also observed that the final concentrations of trisulfide compounds were a consistent percentage of their respective disulfide concentrations, measured at a particular time point. For instance, in the Red Bunching onions, the concentration of DPDS was 10-fold higher compared to the concentration of DPTS upon the application of high-PEF treatment measured at T24. Similar to the other volatile compounds detected in the Yellow Sweet Spanish bulb onions, the highest concentrations of trisulfides were obtained upon the application of medium-PEF treatment, with a decrease at high-PEF intensities.

**Figure 6:**
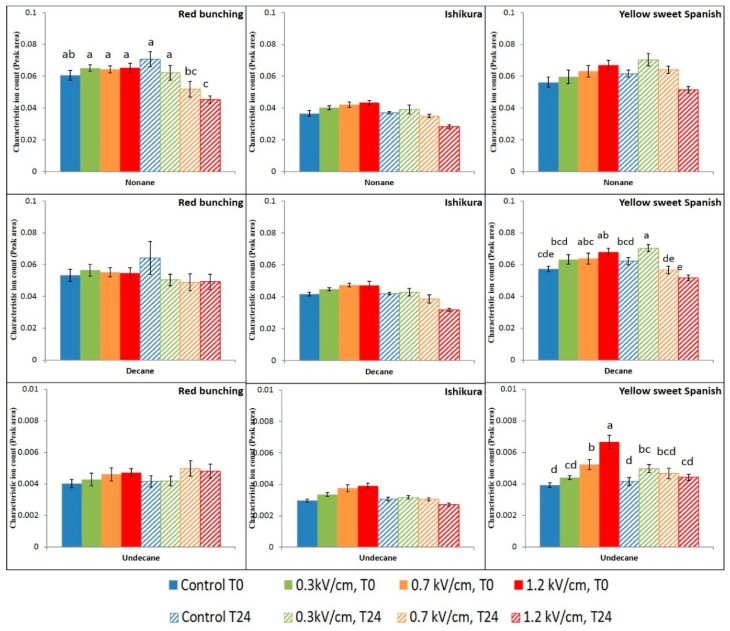
Mean (*n* = 8) ion counts of alkanes (nonane, decane, and undecane) in control and PEF-treated onion samples. Bars with different letters indicate statistically significant difference (Fisher’s LSD, *p* < 0.05). Error bars represent standard error of the mean.

PEF treatment had a limited effect on the concentrations of alkanes (nonane, decane, and undecane) detected in the onion headspace (Figure 6). It is suspected that the formation of alkanes are due to the fragmentation of substrate molecules (non-volatile precursors) or thiosulfinate molecules upon PEF treatment intensities [28,35]. Moreover, the biosynthetic pathways of alkanes and their role in flavour production remains largely unknown.

### 3.4. Limitations

In this study, the PEF treatment of intact onions were studied at electric field strengths lower than 2 kV/cm. Future research could investigate the impact of other PEF parameters such as specific energy, frequency, and number of pulses. The changes in volatile compositions in onion cultivars may affect the perception of onion flavour in culinary applications. Further work would be required to understand the relationship between volatile concentrations and flavour perception.

## 4. Conclusions

The study demonstrated that the application of PEF treatment influenced the volatile profile of onions based on the applied electric field strengths. The intensity of PEF treatment was correlated to the differences in the extent of cellular disruption, which resulted in the mixing of enzyme and substrates, thus facilitating reactions to generate the volatile compounds of interest. The impact of PEF treatment on the volatile compounds were found to be consistent between the three onion cultivars tested. However, quantitative differences in the volatile concentrations were observed between the cultivars, correlating to tissue type and maturity. The highest concentrations of volatile sulfur compounds were observed after 24 h incubation following a high-PEF treatment, compared to the untreated samples. Across all three cultivars, the volatile sulfur-containing compounds such as DPDS, PPrDS, DPTS, MPDS, PPrTS, and MPTS, were found to be significantly affected by PEF treatment intensities and could act as markers of cell membrane damage. This study also suggests that PEF treatment could be tailored for different applications based on the specific fruit and vegetable tissue. Moreover, these volatile compounds could also be used as markers to detect damage or deterioration in fruit and vegetables tissues.

## Figures and Tables

**Table 1:** Identification of volatile compounds in control and pulsed electric field (PEF)-treated onion cultivars.

CAS Number	Volatile Compounds (Abbreviations)	M.W	R.T ^a^ (min)	Calculated RI ^b^	Lit RI ^c^	E.I. ^d^
111-84-2	Nonane	128	6.9	895	900	128
67-56-1	Methanol	32	7.2	910	920	32
124-18-5	Decane	142	9.6	1007	1000	142
1120-21-4	Undecane	156	12.9	1112	1100	156
623-36-9	2-Methyl-2-pentenal (2M2P)	98	15.5	1180	1160	98
2179-60-4	Methyl propyl disulfide (MPDS)	122	17.6	1257	1224	122/80
32157-29-2	Propanethial S-oxide (PSO)	90	17.7	1257	-	90
638-02-8	Dimethyl thiophene (DMT)	112	18.4	1286	1253	111
5905-47-5	Methyl propenyl disulfide (MPrDS)	120	19.4	1316	1327	120
629-19-6	Dipropyl disulfide (DPDS)	150	21.9	1400	1370	150/108
5905-46-4	Propyl propenyl disulfide (PPrDS)	148	23.4	1460	1428	148
17619-36-2	Methyl propyl trisulfide (MPTS)	154	25.3	1533	1516	154/112
2217-02-9	Fenchyl alcohol (IS) ^e^	81	26	1593	1574	81
6028-61-1	Dipropyl trisulfide (DPTS)	182	27.7	1700	1662	182/75
	Propenyl propyl thiosulfinate (PrPthiosulfinate)	180	28.6	1770	-	180
33922-73-5	Propyl propenyl trisulfide (PPrTS)	180	29.2	1817	-	180/74

^a^ Retention time. ^b^ Retention index on a WAX column, calculated in relation to the retention time of n-alkane (C8–C30) series. ^c^ Literature RI values from [21,22]. ^d^ Extracted ion used for quantification. ^e^ IS—internal standard.

## Supplementary Materials

The following are available online at http://www.mdpi.com/2304-8158/7/11/183/s1. Table S1: Volatile compounds in control and PEF-treated samples of Red Bunching onions (immediately after PEF and 24 h after PEF), Table S2. Volatile compounds in control and PEF-treated samples of Ishikura onions (immediately after PEF and 24 h after PEF), Table S3. Volatile compounds in control and PEF-treated samples of Yellow sweet Spanish onions (immediately after PEF and 24 h after PEF), Table S4. Results of ANOVA representing the volatile compounds in onion varieties upon PEF treatment.

## References

[1] Doevenspeck H. (1961). Influencing cells and cell walls by electrostatic impulses. Meat Ind..

[2] Raso J., Heinz V. (2006). Pulsed Electric Fields Technology for the Food Industry.

[3] Raso J., Barbosa-Cánovas G.V. (2003). Nonthermal preservation of foods using combined processing techniques. Crit. Rev. Food Sci. Nutr..

[4] Knorr D., Ade-Omowaye B.I., Heinz V. (2002). Nutritional improvement of plant foods by non-thermal processing. Proc. Nutr. Soc..

[5] Lebovka N.I., Praporscic I., Vorobiev E. (2004). Effect of moderate thermal and pulsed electric field treatments on textural properties of carrots, potatoes and apples. Innov. Food Sci. Emerg. Technol..

[6] Bazhal M.I., Lebovka N.I., Vorobiev E. (2003). Optimisation of pulsed electric field strength for electroplasmolysis of vegetable tissues. Biosystems Eng..

[7] Asavasanti S., Ersus S., Ristenpart W., Stroeve P., Barrett D.M. (2010). Critical electric field strengths of onion tissues treated by pulsed electric fields. J. Food Sci..

[8] Fincan M., DeVito F., Dejmek P. (2004). Pulsed electric field treatment for solid–liquid extraction of red beetroot pigment. J. Food Eng..

[9] Lebovka N.I., Shynkaryk N.V., Vorobiev E. (2007). Pulsed electric field enhanced drying of potato tissue. J. Food Eng..

[10] Janositz A., Semrau J., Knorr D. (2011). Impact of PEF treatment on quality parameters of white asparagus (*Asparagus officinalis* L.). Innov. Food Sci. Emerg. Technol..

[11] Knorr D., Angersbach A. (1998). Impact of high-intensity electric field pulses on plant membrane permeabilization. Trends Food Sci. Technol..

[12] Angersbach A., Heinz V., Knorr D. (2000). Effects of pulsed electric fields on cell membranes in real food systems. Innov. Food Sci. Emerg. Technol..

[13] Ben Ammar J., Lanoisellé J.L., Lebovka N.I., Van Hecke E., Vorobiev E. (2011). Impact of a pulsed electric field on damage of plant tissues: effects of cell size and tissue electrical conductivity. J. Food Sci..

[14] Faridnia F., Burritt D.J., Bremer P.J., Oey I. (2015). Innovative approach to determine the effect of pulsed electric fields on the microstructure of whole potato tubers: Use of cell viability, microscopic images and ionic leakage measurements. Food Res. Int..

[15] Liu T., Dodds E., Leong S.Y., Eyres G.T., Burritt D.J., Oey I. (2017). Effect of pulsed electric fields on the structure and frying quality of “kumara” sweet potato tubers. Innov. Food Sci. Emerg. Technol..

[16] Griffiths G., Trueman L., Crowther T., Thomas B., Smith B. (2002). Onions-A global benefit to health. Phytother. Res..

[17] Selby C., Galpin I.J., Collin H.A. (1979). Comparison of the onion plant (*Allium cepa*) and onion tissue culture. I. Alliinase activity and flavour precursor compounds. New Phytol..

[18] Bacon J.R., Moates G.K., Ng A., Rhodes M.J., Smith A.C., Waldron K.W. (1999). Quantitative analysis of flavour precursors and pyruvate levels in different tissues and cultivars of onion (*Allium cepa*). Food Chem..

[19] Block E. (1985). The chemistry of garlic and onions. Sci. Am..

[20] Mondy N., Duplat D., Christides J.P., Arnault I., Auger J. (2002). Aroma analysis of fresh and preserved onions and leek by dual solid-phase microextraction–liquid extraction and gas chromatography–mass spectrometry. J. Chromatogr. A.

[21] Iranshahi M. (2012). A review of volatile sulfur-containing compounds from terrestrial plants: biosynthesis, distribution and analytical methods. J. Essent. Oil Res..

[22] Bianchi F., Careri M., Mangia A., Musci M. (2007). Retention indices in the analysis of food aroma volatile compounds in temperature-programmed gas chromatography: Database creation and evaluation of precision and robustness. J. Sep. Sci..

[23] Lee S.U., Lee J.H., Choi S.H., Lee J.S., Ohnisi-Kameyama M., Kozukue N., Levin C.E., Friedman M. (2008). Flavonoid content in fresh, home-processed, and light-exposed onions and in dehydrated commercial onion products. J. Agric. Food Chem..

[24] Block E. (1992). The organosulfur chemistry of the genus Allium-implications for the organic chemistry of sulfur. Angew. Chem. Intern. Ed..

[25] Block E., Putman D., Zhao S.H. (1992). Allium chemistry: GC-MS analysis of thiosulfinates and related compounds from onion, leek, scallion, shallot, chive, and Chinese chive. J. Agric. Food Chem..

[26] Schmidt N.E., Santiago L.M., Eason H.D., Dafford K.A., Grooms C.A., Link T.E., Manning D.T., Cooper S.D., Keith R.C., Chance W.O. (1996). Rapid extraction method of quantitating the lachrymatory factor of onion using gas chromatography. J. Agric. Food Chem..

[27] Eady C.C., Kamoi T., Kato M., Porter N.G., Davis S., Shaw M., Kamoi A., Imai S. (2008). Silencing onion lachrymatory factor synthase causes a significant change in the sulfur secondary metabolite profile. Plant Physiol..

[28] Kuo M.C., Ho C.T. (1992). Volatile constituents of the distilled oils of welsh onions (*Allium fistulosum* L. variety maichuon) and scallions (*Allium fistulosum* L. variety caespitosum). J. Agric. Food Chem..

[29] Ersus S., Oztop M.H., McCarthy M.J., Barrett D.M. (2010). Disintegration efficiency of pulsed electric field induced effects on onion (*Allium cepa* L.) tissues as a function of pulse protocol and determination of cell integrity by H-NMR relaxometry. J. Food Sci..

[30] Maskooki A., Eshtiaghi M.N. (2012). Impact of pulsed electric field on cell disintegration and mass transfer in sugar beet. Food Bioprod. Process..

[31] Villière A., Le Roy S., Fillonneau C., Guillet F., Falquerho H., Boussely S., Prost C. (2015). Evaluation of aroma profile differences between sué, sautéed, and pan-fried onions using an innovative olfactometric approach. Flavour.

[32] Tsuge K., Kataoka M., Seto Y. (2002). Determination of S-methyl-, S-propyl-, and S-propenyl-l-cysteine sulfoxides by gas chromatography-mass spectrometry after tert-butyldimethylsilylation. J. Agric. Food Chem..

[33] Kopsell D.E., Randle W.M. (1999). Changes in the S-alk(en)yl cysteine sulfoxides and their biosynthetic intermediates during onion storage. J. Am. Soc. Hortic. Sci..

[34] Randle W.M. (2007). Onion Flavor Chemistry and Factors Influencing Flavor Intensity. Spices.

[35] Yusuf O.K., Bewaji C.O. (2011). Evaluation of essential oils composition of methanolic *Allium sativum* extract on *Trypanosoma brucei* infected rats. Res. Pharm. Biotech..

