# Supplementary material for: Impact of Pulsed Electric Fields on the Volatile Compounds Produced in Whole Onions (Allium cepa and Allium fistulosum)
Source: Foods. 2018 Nov 7;7(11):183. doi: 10.3390/foods7110183 (PMC6262602; doi:10.3390/foods7110183)
Supplement: Supplementary file 1 [file foods-07-00183-s001.pdf]

Table S1. Volatile compounds in control and PEF-treated samples of Red Bunching onions (immediately after PEF and 24 h after PEF).

Cultivar	Compound	Mean ± SEM*							
		T0				T24			
		Control	Low PEF	Medium PEF	High PEF	Control	Low PEF	Medium PEF	High PEF
Red Bunching	Nonane	0.61 ± 0.01 ^{ab}	0.065 ± 0.02 ^{a}	0.064 ± 0.02 ^{a}	0.07 ± 0.003 ^{a}	0.07 ± 0.01 ^{a}	0.06 ± 0.01 ^{a}	0.05 ± 0.01 ^{bc}	0.05 ± 0.01 ^{c}
	Decane	0.05 ± 0.004 ^{ab}	0.06 ± 0.004 ^{ab}	0.06 ± 0.003 ^{ab}	0.06 ± 0.01 ^{ab}	0.064 ± 0.01 ^{a}	0.05 ± 0.01^{ab}	0.05 ± 0.01^{b}	0.05 ± 0.001 ^{b}
	Undecane	0.004 ±0.001 ^{b}	0.004 ± 0.001 ^{a}	0.01 ± 0.001 ^{a}	0.01 ± 0.001 ^{a}	0.004 ± 0.001 ^{a}	0.004 ± 0.001 ^{a}	0.01 ± 0.001 ^{a}	0.01 ± 0.001 ^{a}
	PSO	0.01 ± 0.01 ^{cd}	0.02 ± 0.01 ^{b}	0.02 ± 0.002 ^{ab}	0.023 ± 0.002 ^{a}	0.01 ± 0.01 ^{cd}	0.02 ± 0.03 ^{bc}	0.02 ± 0.001 ^{bc}	0.01 ± 0.001 ^{d}
	2M2P	0.04 ± 0.01 ^{bc}	0.05 ± 0.01 ^{a}	0.05 ± 0.002 ^{ab}	0.06 ± 0.003 ^{a}	0.05 ± 0.01 ^{ab}	0.04 ± 0.01^{cd}	0.04 ± 0.01^{cd}	0.03 ± 0.01^{d}
	DMT	0.2 ± 0.01 ^{cd}	0.24 ± 0.01^{a}	0.21 ± 0.01^{abc}	0.22 ± 0.01 ^{abc}	0.2 ± 0.01 ^{bc}	0.23 ± 0.01 ^{ab}	0.21 ± 0.01^{abc}	0.2 ± 0.02 ^{c}
	DPDS	3.2 ± 0.5 ^{d}	9.52 ± 0.6 ^{c}	9.9 ± 0.5 ^{c}	10.8 ± 0.9 ^{c}	4.02 ± 0.5 ^{d}	22.1 ± 3.3 ^{b}	28.4 ± 2.6 ^{a}	28.8 ± 2.3 ^{a}
	MPDS	0.041 ± 0.01^{d}	0.123 ± 0.01 ^{cd}	0.114 ± 0.01^{cd}	0.19 ± 0.02 ^{c}	0.04 ± 0.02 ^{d}	0.18 ± 0.02 ^{c}	0.33 ± 0.02 ^{b}	0.6 ± 0.1^{a}
	MPrDS	0.04 ± 0.01 ^{d}	0.05 ± 0.01 ^{d}	0.05 ± 0.01 ^{cd}	0.06 ± 0.01 ^{bc}	0.04 ± 0.01 ^{d}	0.07 ± 0.01 ^{abc}	0.08 ± 0.01 ^{ab}	0.08 ± 0.01 ^{a}
	PrPTs	0.01 ± 0.01 ^{ef}	0.012 ± 0.01 ^{def}	0.02 ± 0.02 ^{de}	0.03 ± 0.02 ^{cd}	0.01 ± 0.01 ^{f}	0.04 ± 0.01^{c}	0.1 ± 0.01 ^{b}	0.11 ± 0.02 ^{a}
	PPrDS	1.4 ± 0.1 ^{d}	3.9 ± 0.3 ^{b}	4.4 ± 0.26 ^{b}	5.7 ± 0.5 ^{a}	1.26 ± 0.1 ^{d}	2.85 ± 0.2 ^{c}	4.2 ± 0.3 ^{b}	5.4 ± 0.3 ^{a}
	MPTS	0.002± 0.001^{d}	0.003 ± 0.002 ^{d}	0.003 ± 0.002 ^{d}	0.01 ± 0.001 ^{d}	0.002 ± 0.001 ^{d}	0.02 ± 0.003^{c}	0.03 ± 0.003^{b}	0.05 ± 0.004 ^{a}
	DPTS	0.1 ± 0.02 ^{cd}	0.18 ± 0.02 ^{c}	0.18 ± 0.02 ^{c}	0.26 ± 0.03 ^{c}	0.08 ± 0.01^{cd}	1.58 ± 0.2 ^{b}	2.78 ± 0.44 ^{a}	3.3 ± 0.3 ^{a}
	PPrTS	0.01 ± 0.001 ^{d}	0.01 ± 0.001 ^{d}	0.01 ± 0.001 ^{d}	0.01 ± 0.001 ^{d}	0.01 ± 0.001 ^{d}	0.04 ± 0.004 ^{c}	0.06 ± 0.01 ^{b}	0.08 ± 0.01 ^{a}

^{a,b,c} Different letter within the same row differ significantly using Fisher's LSD post hoc test (p <0.001). * Ratio to internal standard (0.1 ppm Fenchyl alcohol); Low PEF = 0.3 kV/cm, Med PEF = 0.7 kV/cm, High PEF = 1.2 kV/cm.

Table S2. Volatile compounds in control and PEF-treated samples of Ishikura onions (immediately after PEF and 24 h after PEF).

Cultivar	Compound	Mean ± SEM*							
		T0				T24			
		Control	Low PEF	Medium PEF	High PEF	Control	Low PEF	Medium PEF	High PEF
	Nonane	0.04 ± 0.002^{bc}	0.04 ± 0.001 ^{ab}	0.04 ± 0.002 ^{a}	0.04 ± 0.002 ^{a}	0.04 ± 0.001 ^{bc}	0.04 ±0.003^{abc}	0.04± 0.001 ^{c}	0.03 ±0.001 ^{d}
	Decane	0.04± 0.001^{bc}	0.05 ± 0.001^{ab}	0.05 ± 0.001^{a}	0.05 ± 0.003 ^{a}	0.042 ± 0.001 ^{bc}	0.04 ± 0.002 ^{bc}	0.04 ± 0.003 ^{c}	0.03 ± 0.001^{d}
	Undecane	0.003±0.001^{cd}	0.003 ± 0.001 ^{b}	0.004±0.002 ^{a}	0.004± 0.001 ^{a}	0.003±0.001 ^{bcd}	0.003 ± 0.001 ^{bc}	0.003 ± 0.001^{bcd}	0.003 ± 0.001^{d}
	PSO	0.006 ±0.001 ^{c}	0.007 ± 0.001 ^{b}	0.008±0.001 ^{a}	0.008±0.001 ^{a}	0.006±0.001 ^{c}	0.007± 0.001 ^{b}	0.007± 0.001 ^{bc}	0.006±0.001 ^{bc}
	2M2P	0.04 ± 0.003^{b}	0.05 ±0.003 ^{b}	0.05±0.003 ^{ab}	0.053 ± 0.003 ^{a}	0.05± 0.003 ^{b}	0.042 ± 0.001 ^{b}	0.032± 0.001 ^{c}	0.02± 0.0007 ^{d}
	DMT	0.19 ± 0.01 ^{a}	0.2 ± 0.01 ^{a}	0.21 ± 0.01 ^{a}	0.21 ± 0.01 ^{a}	0.20± 0.01 ^{a}	0.211 ± 0.01 ^{a}	0.2 ± 0.01 ^{a}	0.19 ± 0.01 ^{a}
**Ishikura**	DPDS	1.42 ± 0.04 ^{e}	3.6 ± 0.1 ^{d}	4.3 ± 0.1 ^{d}	4.5 ± 0.24 ^{d}	1.2 ± 0.1 ^{e}	18.5 ± 1.1 ^{c}	23.5 ± 0.7 ^{b}	25.6 ± 0.7 ^{a}
	MPDS	0.04 ± 0.001 ^{d}	0.31 ± 0.01 ^{c}	0.37 ± 0.02 ^{c}	0.41 ± 0.01 ^{c}	0.04± 0.002 ^{d}	0.4 ± 0.02 ^{c}	1.5 ± 0.07 ^{b}	2.04 ± 0.07 ^{a}
	MPrDS	0.03 ± 0.004^{bc}	0.032±0.004 ^{abc}	0.04±0.003 ^{ab}	0.042 ± 0.002 ^{a}	0.03± 0.005 ^{c}	0.032±0.003 ^{abc}	0.04± 0.0045 ^{abc}	0.04 ± 0.003 ^{a}
	PrPTs	0.01 ± 0.001 ^{e}	0.022 ± 0.003 ^{d}	0.031 ±0.002^{c}	0.035 ± 0.001^{c}	0.008± 0.001^{e}	0.04 ± 0.001 ^{c}	0.12± 0.006 ^{b}	0.162± 0.006 ^{a}
	PPrDS	1.21 ± 0.1 ^{e}	3.6 ± 0.2 ^{b}	3.94 ± 0.2 ^{b}	4.5 ± 0.2 ^{a}	1.04 ± 0.07 ^{e}	1.7 ± 0.08 ^{d}	3.13 ± 0.2 ^{c}	3.7 ± 0.13 ^{b}
	MPTS	0.004 ±0.001 ^{c}	0.006±0.001 ^{c}	0.01 ± 0.001 ^{c}	0.011± 0.001 ^{c}	0.004± 0.0002 ^{c}	0.012± 0.001 ^{c}	0.092± 0.006 ^{b}	0.145± 0.02 ^{a}
	DPTS	0.061 ± 0.01 ^{d}	0.132 ± 0.005 ^{d}	0.16± 0.011 ^{d}	0.19 ± 0.01 ^{d}	0.023± 0.002 ^{d}	0.43 ± 0.03 ^{c}	3.9 ± 0.14 ^{b}	4.5 ± 0.1 ^{a}
	PPrTS	0.01 ±0.001 ^{c}	0.012 ± 0.001 ^{c}	0.015± 0.001 ^{c}	0.016 ± 0.001 ^{c}	0.008± 0.001 ^{c}	0.12 ± 0.008 ^{b}	0.15 ± 0.008 ^{a}	0.16 ± 0.01 ^{a}

[a,b,c] Different letter within the same row differ significantly using Fisher's LSD post hoc test (*p* <0.001). * Ratio to internal standard (0.1 ppm Fenchyl alcohol); Low PEF = 0.3 kV/cm, Med PEF = 0.7 kV/cm, High PEF = 1.2 kV/cm.

Table S3. Volatile compounds in control and PEF-treated samples of Yellow sweet Spanish onions (immediately after PEF and 24 h after PEF).

Cultivar	Compound	Mean ± SE*							
		T0				T24			
		Control	Low PEF	Medium PEF	High PEF	Control	Low PEF	Medium PEF	High PEF
Yellow sweet Spanish	Nonane	0.06 ± 0.003^{cd}	0.06 ± 0.004^{bcd}	0.06 ± 0.004^{abc}	0.067 ± 0.003^{ab}	0.062 ±0.002^{abc}	0.07 ± 0.004^{a}	0.06 ± 0.002^{abc}	0.05 ± 0.002^{d}
	Decane	0.06±0.002^{cde}	0.063±0.003^{bcd}	0.064±0.003^{abc}	0.07±0.002^{ab}	0.062±0.0022^{bcd}	0.07 ± 0.002^{a}	0.06 ± 0.002^{de}	0.05±0.002^{e}
	Undecane	0.004±0.0002^{d}	0.004±0.0001^{cd}	0.005±0.0003^{b}	0.007±0.0004^{a}	0.004±0.0003^{d}	0.005±0.0003^{bc}	0.005±0.0003^{bcd}	0.004±0.0002^{cd}
	PSO	0.02 ± 0.001^{c}	0.033 ± 0.003^{b}	0.04 ± 0.003^{a}	0.04 ± 0.003^{a}	0.019 ± 0.001^{c}	0.03 ± 0.002^{b}	0.03 ± 0.001^{cd}	0.032 ±0.0024^{b}
	2M2P	0.037±0.002^{cd}	0.062 ± 0.004^{b}	0.06 ± 0.005^{b}	0.08 ± 0.01^{a}	0.034±0.004^{cd}	0.042±0.005^{c}	0.03±0.004^{cd}	0.03 ± 0.005^{d}
	DMT	0.18 ± 0.01^{abc}	0.2 ± 0.01^{ab}	0.22 ± 0.02^{a}	0.17 ± 0.024^{bc}	0.20 ± 0.023^{bc}	0.2 ± 0.01^{bc}	0.16 ± 0.02^{bc}	0.15 ± 0.01^{c}
	DPDS	2.68 ± 0.5 ^{d}	4.93 ± 0.22 ^{d}	5.83 ± 0.3 ^{d}	4.2 ± 0.3 ^{d}	3.11 ± 0.2 ^{d}	30.5 ± 1.4^{b}	33.3 ± 1.1^{a}	27.7 ± 1.14 ^{c}
	MPDS	0.01 ± 0.002 ^{d}	0.13 ± 0.03 ^{c}	0.16 ± 0.012 ^{c}	0.08 ± 0.014^{cd}	0.014 ±0.003^{d}	0.32 ± 0.04 ^{b}	0.56 ± 0.06 ^{a}	0.4 ± 0.04 ^{b}
	MPrDS	0.02 ± 0.002 ^{c}	0.023 ± 0.002^{bc}	0.03 ± 0.002^{b}	0.021 ± 0.001^{c}	0.02 ± 0.003^{c}	0.03 ± 0.002^{b}	0.04 ± 0.003^{a}	0.03 ± 0.001^{b}
	PrPTs	0.02 ± 0.004^{c}	0.058 ± 0.004^{c}	0.08 ± 0.003 ^{c}	0.05 ± 0.003 ^{c}	0.02 ± 0.002 ^{c}	0.4 ± 0.02 ^{b}	0.51 ± 0.046 ^{a}	0.34 ± 0.04^{b}
	PPrDS	1.9 ± 0.2 ^{e}	3.2 ± 0.3 ^{d}	3.4 ± 0.19 ^{cd}	2.74 ± 0.2 ^{d}	1.94 ± 0.13 ^{e}	3.9 ± 0.25 ^{bc}	4.93 ± 0.3 ^{a}	4.41 ± 0.4 ^{ab}
	MPTS	0.003±0.0003^{d}	0.006± 0.0003^{d}	0.006 ± 0.001^{d}	0.034 ±0.0003^{d}	0.003±0.0003^{d}	0.03 ± 0.003 ^{c}	0.08 ± 0.007 ^{a}	0.05 ± 0.005 ^{b}
	DPTS	0.08 ± 0.013^{c}	0.3 ± 0.03^{c}	0.37 ± 0.04^{c}	0.26 ± 0.04^{c}	0.09 ± 0.01^{c}	6.3 ± 0.4 ^{a}	6.62 ± 0.25 ^{a}	5.13 ± 0.44^{b}
	PPrTS	0.013 ± 0.0013 ^{c}	0.018 ± 0.0012^{c}	0.025 ± 0.002 ^{c}	0.018 ± 0.003 ^{c}	0.012 ± 0.001^{c}	0.17 ± 0.03 ^{b}	0.27 ± 0.03 ^{a}	0.19 ± 0.02 ^{b}

^{a,b,c} Different letter within the same row differ significantly using Fisher's LSD post hoc test (p <0.001).
* Ratio to internal standard (0.1 ppm Fenchyl alcohol).
;Low PEF = 0.3 kV/cm, Med PEF = 0.7 kV/cm, High PEF = 1.2 kV/cm.

Commented [t1]: Please confirm if the * should be here.

Commented [Sze2R1]: Yes. This is the correct location.
The explanation of * symbol is explain in the footnote

Table S4. Results of ANOVA representing the volatile compounds in onion varieties upon PEF treatment.

Volatile compounds	Treatment	Timepoint	Treatment *Timepoint	Treatment	Timepoint	Treatment *Timepoint	Treatment	Timepoint	Treatment *Timepoint
	**Ishikura**			**Red Bunching**			**Yellow Sweet Spanish bulbs**		
Nonane	F = 2.5<br>$p = 0.072$	F = 26.4<br>$p < 0.001$	F = 9.9<br>$p < 0.001$	F = 3.5<br>$p = 0.02$	F = 6.1<br>$p = 0.02$	F = 6.5<br>$p = 0.001$	F = 1.9<br>$p = 0.13$	F = 0.1<br>$p = 0.87$	F = 6.4<br>$p = 0.001$
Decane	F = 2.4<br>$p = 0.076$	F = 27.7<br>$p < 0.001$	F = 8.3<br>$p < 0.001$	F = 0.7<br>$p = 0.56$	F = 0.2<br>$p = 0.64$	F = 1.2<br>$p = 0.32$	F = 4.1<br>$p = 0.01$	F = 2.7<br>$p = 0.103$	F = 10.1<br>$p < 0.001$
Undecane	F = 2.8<br>$p = 0.055$	F = 27.5<br>$p < 0.001$	F = 9.1<br>$p < 0.001$	F = 0.5<br>$p = 0.61$	F = 2.1<br>$p = 0.16$	F = 1.1<br>$p = 0.4$	F = 10.5<br>$p < 0.001$	F = 6.8<br>$p = 0.012$	F = 10.8<br>$p < 0.001$
2-Methyl-2-pentenal	F = 8.1<br>$p < 0.001$	F = 68.7<br>$p < 0.001$	F = 27.3<br>$p < 0.001$	F = 0.1<br>$p = 0.94$	F = 27.4<br>$p < 0.001$	F = 8.6<br>$p = 0.001$	F = 4.2<br>$p = 0.01$	F = 54.8<br>$p < 0.001$	F = 8.3<br>$p < 0.001$
Dimethyl thiophene	F = 0.4<br>$p = 0.78$	F = 0.3<br>$p = 0.61$	F = 1.7<br>$p = 0.18$	F = 4.2<br>$p = 0.01$	F = 0.9<br>$p = 0.34$	F = 0.5<br>$p = 0.65$	F = 1.3<br>$p = 0.27$	F = 6.8<br>$p = 0.012$	F = 1.5<br>$p = 0.23$
Propanethial S-oxide	F = 8.5<br>$p < 0.001$	F = 12.9<br>$p < 0.001$	F = 5.4<br>$p = 0.003$	F = 5.2<br>$p = 0.003$	F = 15.9<br>$p < 0.001$	F = 6.6<br>$p < 0.001$	F = 30.9<br>$p < 0.001$	F = 19.9<br>$p < 0.001$	F = 3.6<br>$p = 0.02$
Dipropyl disulfide	F = 298.9<br>$p < 0.001$	F = 1451.4<br>$p < 0.001$	F = 179.6<br>$p < 0.001$	F = 32.5<br>$p < 0.001$	F = 89.2<br>$p < 0.001$	F = 9.7<br>$p < 0.001$	F = 167.2<br>$p < 0.001$	F = 1236.2<br>$p < 0.001$	F = 133.4<br>$p < 0.001$
Methyl propyl disulfide	F = 414.8<br>$p < 0.001$	F = 723.0<br>$p < 0.001$	F = 229.3<br>$p < 0.001$	F = 43.4<br>$p < 0.001$	F = 56.9<br>$p < 0.001$	F = 17.3<br>$p < 0.001$	F = 51.9<br>$p < 0.001$	F = 128.5<br>$p < 0.001$	F = 18.3<br>$p < 0.001$
Methyl propenyl disulfide	F = 5.2<br>$p = 0.003$	F = 0.2<br>$p = 0.68$	F = 0.01<br>$p = 0.99$	F = 13.9<br>$p < 0.001$	F = 17.1<br>$p < 0.001$	F = 1.7<br>$p = 0.18$	F = 14.3<br>$p < 0.001$	F = 11.4<br>$p < 0.001$	F = 1.6<br>$p = 0.21$
Propenyl propyl thiosulfinate	F = 290.9<br>$p < 0.001$	F = 558.3<br>$p < 0.001$	F = 156.9<br>$p < 0.001$	F = 40.3<br>$p < 0.001$	F = 84.3<br>$p < 0.001$	F = 15.9<br>$p < 0.001$	F = 50.9<br>$p < 0.001$	F = 269.2<br>$p < 0.001$	F = 32.5<br>$p < 0.001$
Propenyl propyl disulfide	F = 147.5<br>$p < 0.001$	F = 74.2<br>$p < 0.001$	F = 11.4<br>$p < 0.001$	F = 94.8<br>$p < 0.001$	F = 5.6<br>$p = 0.02$	F = 1.5<br>$p = 0.22$	F = 30.<br>$p < 0.001$	F = 31.8<br>$p < 0.001$	F = 4.6<br>$p = 0.006$
Dipropyl trisulfide	F = 761.3<br>$p < 0.001$	F = 2355.4<br>$p < 0.001$	F = 700.8<br>$p < 0.001$	F = 29.2<br>$p < 0.001$	F = 142.3<br>$p < 0.001$	F = 21.3<br>$p < 0.001$	F = 105.2<br>$p < 0.001$	F = 774.4<br>$p < 0.001$	F = 89.5<br>$p < 0.001$
Methyl propyl trisulfide	F = 61.9<br>$p < 0.001$	F = 151.6<br>$p < 0.001$	F = 51.5<br>$p < 0.001$	F = 57.6<br>$p < 0.001$	F = 238.6<br>$p < 0.001$	F = 44.1<br>$p < 0.001$	F = 47.3<br>$p < 0.001$	F = 240.2<br>$p < 0.001$	F = 42.5<br>$p < 0.001$

Commented [t3]: Please explain the symbol * in the footnote.

**Volatile compounds**	**Treatment**	**Timepoint**	**Treatment *Timepoint**	**Treatment**	**Timepoint**	**Treatment *Timepoint**	**Treatment**	**Timepoint**	**Treatment *Timepoint**
	**Ishikura**			**Red Bunching**			**Yellow Sweet Spanish bulbs**		
Propenyl propyl trisulfide	F = 78.1 $p < 0.001$	F = 548.3 $p < 0.001$	F = 64.9 $p < 0.001$	F = 22.3 $p < 0.001$	F = 119.8 $p < 0.001$	F = 16.9 $p < 0.001$	F = 24.9 $p < 0.001$	F = 152.9 $p < 0.001$	F = 20.9 $p < 0.001$

* Indicates the two-way ANOVA interaction between treatment and time point.

Commented [t3]: Please explain the symbol * in the footnote.

Formatted: Highlight
